# Study of Laser-Induced Multi-Exciton Generation and Dynamics by Multi-Photon Absorption in CdSe Quantum Dots

**DOI:** 10.3390/nano14070558

**Published:** 2024-03-22

**Authors:** Peng Zhang, Yimeng Wang, Xueqiong Su, Qiwen Zhang, Mingyu Sun

**Affiliations:** 1 Institute of Photonic Chips, University of Shanghai for Science and Technology, Shanghai 200093, China; 212180335@st.usst.edu.cn (P.Z.); wangyimeng@usst.edu.cn (Y.W.); 212180328@st.usst.edu.cn (Q.Z.); 2 Centre for Artificial-Intelligence Nanophotonics, School of Optical-Electrical and Computer Engineering, University of Shanghai for Science and Technology, Shanghai 200093, China; 3 School of Physics and Optoelectronic Engineering, Beijing University of Technology, Beijing 100124, China; nysxq@bjut.edu.cn

**Keywords:** quantum dots, multi-photon absorption, pump–pulse energy, hot exciton cooling, auger recombination

## Abstract

Multi-exciton generation by multi-photon absorption under low-energy photons can be thought a reasonable method to reduce the risk of optical damage, especially in photoelectric quantum dot (QD) devices. The lifetime of the multi-exciton state plays a key role in the utilization of photon-induced carriers, which depends on the dynamics of the exciton generation process in materials. In this paper, the exciton generation dynamics of the photon absorption under low-frequency light in CdSe QDs are successfully detected and studied by the temporal resolution transient absorption (TA) spectroscopy method. Since the cooling time of hot excitons extends while the rate of auger recombination is accelerated when incident energy is increased, the filling time of defect states is irregular, and exciton generation experiences a transition from single-photon absorption to multi-photon absorption. This result shows how to change the excitation. Optical parameters can prolong the lifetime of excitons, thus fully extracting excitons and improving the photoelectric conversion efficiency of QD optoelectronic devices, which provides theoretical and experimental support for the development of QD optoelectronic devices.

## 1. Introduction

**Figure 1:**
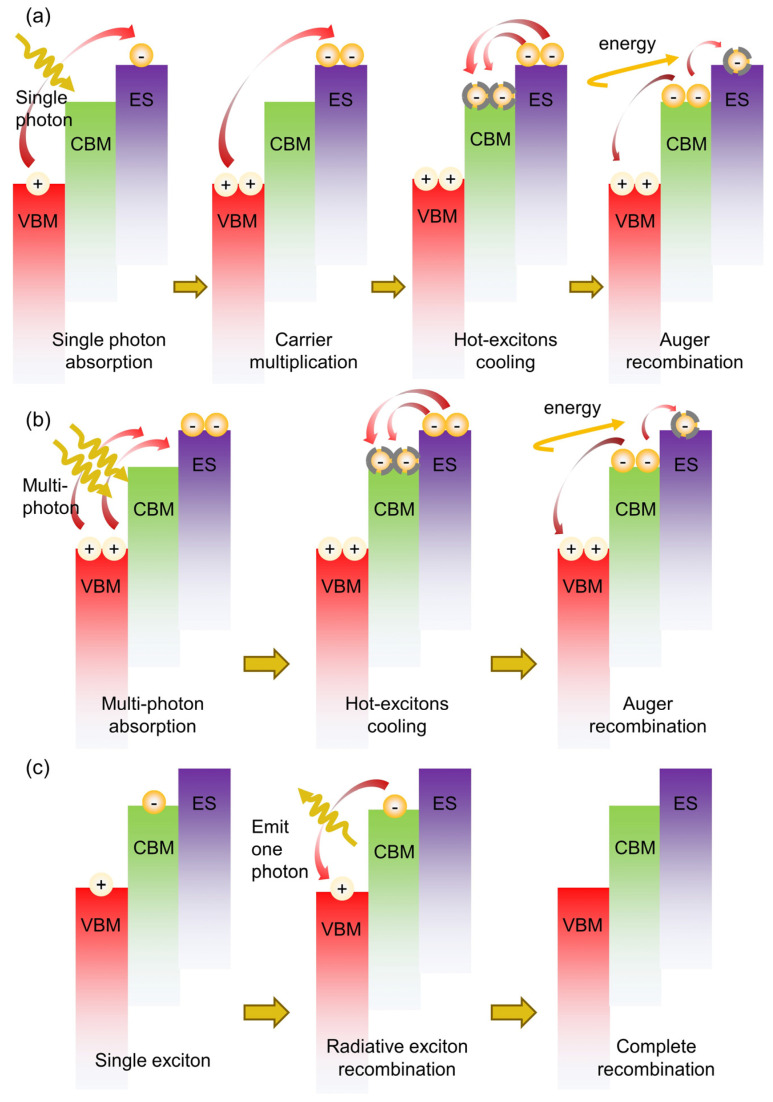
(**a**) Carrier multiplication produces multi-exciton and (**b**) multi-photon absorption produces multi-exciton. (**c**) Single-exciton recombination. VBM: top of valence band; CBM: base of conduction band; ES: excited state.

Recently, quantum dots (QDs) have attracted attention in various applications like solar cells, quantum computing, and light-emitting diodes (LEDs) [1,2,3]. Due to the significant quantum confinement effect, carrier population in QDs can be enhanced under multi-exciton generation by ultrafast laser stimulation, which would effectively improve the photoelectric performance of devices [4]. More often, QDs are prepared using inorganic semiconductors, e.g., Si, CdSe, InP, PbS, etc. Comparatively, the typical II–VI group semiconductor CdSe is reported to have stronger photoelectric conversion efficiency in the visible range, showing potential in luminance, QD-sensitized solar cell devices, and biomedical applications [5,6,7,8,9]. There are two ways of generating multi-exciton states using an ultrafast laser to excite materials. The first method is the production of two or even more electron–hole pairs, i.e., the carrier multiplication effect (CM), by absorbing a high-energy photon [4], which is shown in the sketch of physics process in Figure 1a,c. The spherical symbol at the top of the valence band represents holes, while the spherical symbol at the base of the conduction band and excited state represents electrons. The other method, as shown in Figure 1b,c, is the absorption of two or even more photons by QDs to produce a multi-exciton with strong light irradiation [10]. It should be noted that the multi-photon absorption here is different from the up-conversion type of multi-photon absorption, which involves the multi-photon absorption of photons with an energy between one and two times the bandgap of the material, and the result is also different from that of the up-conversion type. For the up-conversion type, materials absorb multiple photons to generate a single exciton. In this study, QDs absorb multiple photons to generate a multi-exciton. The phrase ‘multi-photon absorption’ in this manuscript can be explained as ‘multi-exciton generation by multi-photon absorption’. More often, the multi-exciton generation process results from the first mechanism, i.e., the generation of a multi-exciton using one photon [11,12,13], which calls for a strong photon energy of more than double the bandgap of the materials, and would potentially cause optical damage like photochromic or a reduction in stability [14]. Alternatively, the two-photon absorption mechanism is proposed, i.e., the generation of excitons via lower-energy photons, to reduce such high-energy damage [15,16].

The exciton dynamics of a coupled multi-exciton after multi-photon absorption in QDs can be induced sequentially as hot exciton cooling, non-radiative recombination of multi-photon (auger recombination) as well as single-exciton radiative recombination [15]. Therefore, extension of multi-photon lifetime is necessary to improve the utilization of photocarriers, thus, the excitons will be effectively parted before the fast auger recombination, and become free moving carriers to be used [17]. For example, in QDs based solar cells, the photoelectric conversion efficiency can be improved by extension of cooling time to fully extract the hot carriers [18]. In QD-based lasers, the suppression of auger recombination will extend the exciton lifetime and improve the population inversion, enhancing the optical gain in the device [19]. Generally, the photoelectric conversion efficiency in QDs is determined by both the hot exciton cooling time as well as the auger recombination rate, which will be influenced by the material composition and scale of QDs, pump photon energy, as well as the pump–pulse energy and sample temperatures. [20,21,22]. The time scale of the two processes ranges approximately from hundreds of femtoseconds (fs) to tens of picoseconds (ps) [23], which calls for fs ultrafast laser pulses to identify the transient process. Mohammed et al. studied the influence of the size effect of PbS QDs on the photon energy threshold to generate the CM effect, and the pump photon energy influence on hot exciton cooling and auger recombination, under high-energy ultrafast laser pumping using transient absorption (TA) spectroscopy with a temporal resolution of 120 fs [24]. The cooling time of hot excitons will be extended with either the increase in pump photon energy or the size of QDs. Liu et al. studied the lifetime of the multi-exciton state and the rate of auger recombination under single-photon absorption in InP/ZnS core/shell QDs pumped by a 400 nm ultrafast laser pulse [25]. Qin et al. studied the exciton dynamics in PbS based on the temporal resolution TA spectroscopy method and found an extension of the hot exciton cooling time with an accelerated rate of auger recombination under increased pump light energy due to the quantum confinement effect [26]. They attributed the CM effect in PbS QDs to the absorption of a single high-energy photon. For comparison, multi-photon absorption can be used as an alternative means of multi-exciton generation in QDs, in which only longer-wavelength photons with lower energy can be acceptable. Therefore, in the multi-photonic absorption process, to reduce the risk of optical damage, prevent the rapid depletion of carriers, and improve the utilization rate, it is necessary to study the dependence of the hot exciton cooling time and auger recombination rate on pump–pulse energy in QDs.

In this paper, the exciton dynamics depending on variable pump–pulse energies were investigated in CdSe QDs pumped by photons with energy from single to double that of the bandgap energy. The carrier transition changes corresponding to the generation and decay of excitons can be detected by ultrafast time-resolved TA spectroscopy. The dynamics of the generation of a multi-exciton via multi-photon absorption process is experimentally studied to explore the mechanism as well as the pump–pulse energy dependence of the hot excitons cooling time and the auger recombination rate.

## 2. Materials and Methods

### 2.1. Synthesis of CdSe QDs

For the selenium (Se) precursor, the NaHSe solution was freshly prepared by dissolving Se powder (1 mmol) and NaBH_4_ (4 mmol) in 4 mL of deionized water, then stirring for several hours at room temperature until the reaction solution seemed clearly transparent or light purple. The NaHSe precursor solution was obtained from the oxidization of Se powder using NaBH_4_, and the empirical formula is exhibited as below:

4NaBH_4_ + 2Se + 7H_2_O = 2NaHSe + Na_2_B_4_O_7_ + 14H_2_O (1)

In the meantime, the Cd(NO_3_)_2_·4H_2_O (0.1 mmol) solution and glutathione (GSH) (0.25 mmol) were dissolved in 25 mL of deionized water for the preparation of the cadmium (Cd) precursor. Then, the NaOH (1 M) solution was mixed into the Cd precursor solution until the pH of solution was 9. Afterward, 100 μL of Se precursor solution was injected rapidly into the Cd precursor solution and refluxed at 100 °C for typical CdSe QD synthesis. The resulting mixture of QD solution was added to isopropanol, followed by centrifugation at 8000 rpm for 10 min. The resulting precipitate was dissolved in deionized water.

### 2.2. Optical Measurement

**Figure 2:**
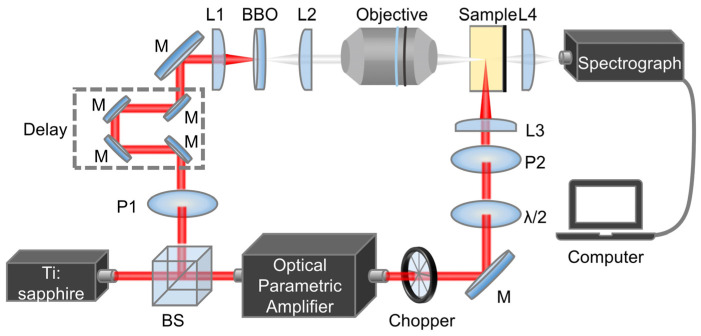
Time-resolved transient absorption spectroscopy system for sample detection. Some symbolic letters are represented as follows: M: mirror; BS: beam splitter; L: convex lens; BBO: β-BaB_2_O_4_; P: Polaroid; λ/2: half-wave plate.

Absorption spectra of the samples were measured by a UV–vis spectrophotometer (Shimadzu-UV-2600i), while the fluorescence spectra of the samples were measured using a high-speed spectrometer (AvoSpec-ULS2048CL-EVO-RS). As shown in Figure 2, the laser source for the time-resolved TA spectroscopy system was a Ti–sapphire amplified laser system (Solstice-Ace, spectrum-physics) with a center wavelength of 800 nm and a repetition frequency of 5 kHz. The incident beam from the light source was split into two beams by a beam splitter. One beam was used as a probe beam by passing through a β-BaB_2_O_4_ (BBO) crystal to produce continuous white light, while another beam was injected into an optical parametric amplifier (OPA), which converted the incident beam into a pump beam in the wavelength range of 245–2600 nm, and the beam energy can be modified by the combination of a half-wave plate and a polarizer. A motor-controlled optical delay line (Newport-DL325) was used to adjust the delay time between the pump beam and the probe beam with a time resolution of 2 fs. The excitation beam was passed through a chopper (Thorlabs-MC1F60), which was modulated to 500 Hz, further allowing the samples to be excited at the frequency by the chopper.

A high-speed spectrometer (AvoSpec-ULS2048CL-EVO-RS) was used to measure the intensity of probe beams for both ‘pumped’ and ‘unpumped’ samples, and to obtain the spectrum of the absorbance difference:

∆A(λ) = −lg[I(λ)_pump_/I(λ)_unpump_] (2)

**Figure 3:**
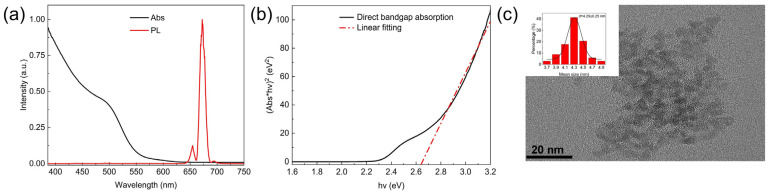
(**a**) Steady state absorption (Abs) spectrum and photoluminescence (PL) spectrum of fabricated CdSe quantum dots (QDs). (**b**) Bandgap calculation based on steady state absorption spectrum. (**c**) Transmission electron microscope (TEM) image of CdSe QDs, the inset is the Particle size distribution analysis.

The setup for signal detection and manipulation were controlled by Labview-2020 software. When the zero-delay point of the pump and probe lights was determined, the absorption spectra were continuously collected by extending the delay line of the system from 0 to 2000 ps. A 340 nm-wavelength pump light was chosen as the source since the photon energy was larger than the bandgap of CdSe QDs (2.64 eV; the bandgap analysis section is shown in Figure 3b) but lower than the threshold of a single photon to produce the CM effect, i.e., high energy of a photon sufficient to produce multiple carriers; thus, only multi-photon absorption needed to be considered for the CM effect during the pump light lamination. The pump–pulse energies of 50 nJ, 100 nJ, 150 nJ, 200 nJ, 500 nJ, 1000 nJ, 1500 nJ, and 2000 nJ were chosen to explore the dependence of the incident energy of pump lights on the CM effect. Experiments on CdSe QDs dissolved in deionized water inside a quartz cuvette were performed.

## 3. Results and Discussion

Before the study of the exciton dynamics, Figure 3a shows the steady state absorption (Abs) spectrum and the photoluminescence (PL) spectrum of CdSe QDs under the continuous white light spectrum probe as the spectral characteristics. Figure 3b shows the bandgap calculation results, which were calculated based on steady state absorption spectrum data in Figure 3a by referring to the theoretical formula of Tauc et al. [27]:

(αhν)^(1/m)^ = B(hν − Ε_g_) (3)

In the formula, α is the absorption coefficient, B and m are constants, h is the Planck constant, ν is the frequency of incident photons, and E_g_ is the material wide bandgap, also known as the bandgap. It can be observed from Figure 3b that the bandgap width of the QDs is about 2.64 eV, which corresponds to a wavelength of 470 nm [27]. Figure 3a also gives the steady state fluorescence spectrum of CdSe, showing the photoluminescence peak wavelength at around 680 nm with a small full width at half maximum (FWHM), no wider than 30 nm. However, there are other peak signals on both sides near 680 nm, and the fluorescence peak at 680 nm is not perfectly symmetrical. This phenomenon is caused by two factors: one is the uneven particle size distribution of QDs, and the other is the presence of defect states in QDs. The optical properties such as absorption and emission spectra can be changed according to various sizes of QDs, which makes QDs widely used in the application of single-photon emitters, biomedicine, and so on [28,29,30]. In order to analyze the size distribution of the QDs, the histogram of the QD size distribution is displayed as an insert in the TEM image of the QDs as shown in Figure 3c. It can be observed that the size distribution of the QDs is relatively uniform, which has limited influence on its absorption and emission spectra without obvious interference in the experimental results of the exciton dynamics. It also indirectly indicates that the prepared CdSe QDs have defect states [31]. According to the photoluminescence spectrum in Figure 3a, the defect energy levels produced by the defect state are around 666 nm and 705 nm, and the ratio of the peak intensity in the photoluminescence spectrum to the intrinsic luminescence intensity of the QDs is about 1:8.

**Figure 4:**
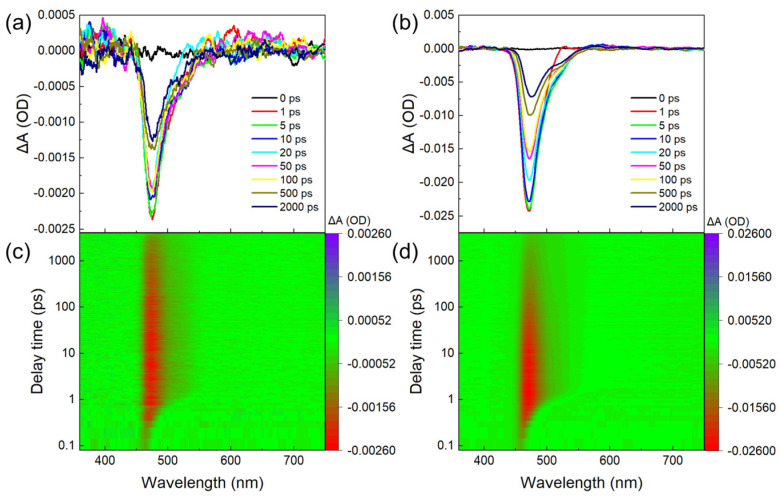
Transmittance diagram of signal evolution over time at 340 nm wavelength, pumped with pulse energy of (**a**) 100 nJ and (**b**) 2000 nJ, respectively. Transmittance isogram evolution over time at 340 nm wavelength, pumped with pulse energy of (**c**) 100 nJ and (**d**) 2000 nJ, respectively.

To study the ultrafast exciton generation and decay kinetics of the CdSe QDs, the temporal resolution TA spectroscopy measurements under various pump–pulse energies were performed. Figure 4 shows the transient absorption spectrum signal diagram of CdSe QDs in the time range of 0 to 2000 ps after being excited by the pump beam. The ground state bleaching (GSB) signal of nine different delay times from 0 ps to 2000 ps between the probe and the pump light under the lower pump–pulse energy of 100 nJ are presented in Figure 4a, together with the contour graph of the transmittance for all continuous variations of the delay time from 0 to 2000 ps in Figure 4c. The negative value marked in red indicates the absorption stage during the whole exciton dynamics process. For comparison, Figure 4b,d gives the GSB and contour graph for higher pump–pulse energy of 2000 nJ. When the valence band electrons in the QDs absorb the energy of the photons and excite to the conduction band, they will fall to the bottom of the conduction band at speeds in the range of hundreds of fs, which shows the hot exciton cooling process after being excited. Since abundant excited electrons are located at the bottom of the conduction band, the lower energy of the probe light is absorbed by the excited samples compared to the ground state samples, thus showing a negative GSB signal in the absorption spectra.

Then, we compared the GSB signals for nine delay times: 0 ps, 1 ps, 4 ps, 10 ps, 20 ps, 50 ps, 100 ps, 500 ps, and 2000 ps under 100 nJ and 2000 nJ incident energies (see Figure 4a,b). For the lower pump–pulse energy of 100 nJ, the bleaching signal happens in the range of 450 nm to 550 nm, with peaks at 470 nm. For the higher pulse energy of 2000 nJ, the bleaching signal also happens in the same range of 450 nm to 550 nm, but with a stronger signal. When the sample is excited under the stronger pump light, more electrons will be excited to the conduction band, reducing the absorption of the probe light. To be noted, in either graph for the 100 nJ or 2000 nJ pump light, the GSB signals increase first and then decrease with the extension of the delay time. During the stage of increasing signal, the electrons were continuously excited to the higher energy state in the conduction band under the influence of the femtosecond laser pulses. With fewer the electrons in the valence band, the energy absorbed will be decreased, increasing the transmittance. After that, the excitons will recombine and fall to the ground state, leading to increased ground state electrons with stronger photon energy absorption, reducing the transmittance. To be noted, the GSB signal is not totally diminished even at the maximum delay time of 2000 ps, showing that the period for exciton radioactive recombination is longer than the set delay time.

Compared with the transient absorption spectrum in the early delay time, the peak position of the bleaching signal in the transient absorption spectrum of the long delay time has a slight red shift, and the red shift is more obvious when the pump–pulse energy is 2000 nJ due to the interaction between excitons. When the interaction force between excitons is gravity, the bandgap of the material decreases, and the spectrum experiences a red shift; otherwise, the bandgap of the material increases, and the spectrum experiences a blue shift. When the pump–pulse energy is high, the spectral shift is more obvious, which is because the interaction between excitons increases with the increase in the number of excitons.

As in the contour graph in Figure 4c,d, for the 100 nJ pump–pulse energy, the GSB signal quickly increases and reaches the peak value within 1 ps, then slightly decays during the next 1 ns. Differently, for the 2000 nJ pulse energy pump light, the GSB signal quickly decays during the next tens of ps after the peak value. This is because when the QDs were illuminated at low intensity by photons with an energy lower than double the bandgap, only a single exciton can be produced by a single photon in the QDs. Few electrons can be accumulated at the bottom of the conduction band under low pump–pulse energy, producing radiative recombination, and the GSB signal decreases slightly and slowly over a periodic range of ns [32]. However, for stronger energies sufficient for the threshold of multi-photon absorption, there is the possibility that some of the QDs will absorb two or multiple photons at one time and produce two or multiple excitons. The coupling of the interaction between excitons leads to a fast auger recombination over a periodic range of ps, and the GSB decays rapidly [33,34]. Therefore, the curves of the exciton dynamics show a slow decay and fast decay process after the excitation of electrons, which can be accordingly attributed to the principle of single-exciton radioactive recombination under a lower pump–pulse energy, and auger recombination between biexcitons under a higher pump–pulse energy. Additionally, for the lower pump–pulse energy, there is no fast decay performance, proving that there is no auger recombination happening, i.e., multi-photon absorption only happens under strong pump energy during the process.

Additionally, by analyzing Figure 4c,d, many issues worth discussing can be discovered. It can be observed that the transient absorption spectrum does not seem to be symmetrical about the peak position of the bleaching signal (470 nm), with some absorbed components between 500 nm and 550 nm. It can be more obviously seen when the pump–pulse energy is 2000 nJ rather than 100 nJ. This phenomenon may be caused by the phonon-assisted transition, or by the absorption of some defect states, or impurity levels. When the pump–pulse energy increases from 100 nJ to 2000 nJ, the position of the bleaching signal band does not change. This shows that the energy level transition of electrons in CdSe QDs will not change with the increase in pump–pulse energy. In the delay time of 1 ps after the pump–pulse excitation, the spectrum gradually spreads from 470 nm to the infrared region due to the dispersion of the probe light on the surface of the material. Since the probe light is white light containing beam components at various wavelengths, the light with different wavelengths reaches the surface of the sample at different times, which will cause dispersion on the surface of the sample.

**Figure 5:**
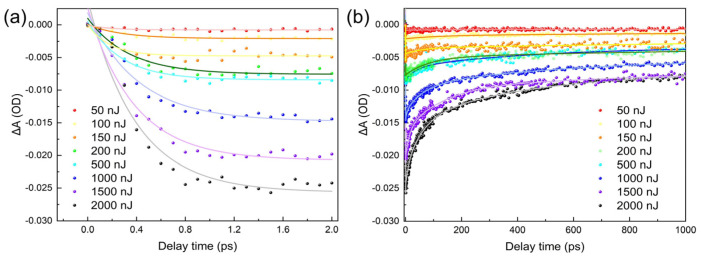
Ground state bleaching (GSB) peak signal evolution tendency under different pump–pulse energies in (**a**) early delay times and (**b**) long delay times. The dots are measured results, and the curves are fittings of measurements.

To show the multi-photon process of exciton dynamics in materials, including the cooling time of hot excitons and the rate of auger recombination depending on the pump–pulse energy, the delay time performance of the GSB signal at 470 nm with eight pump–pulse energies of 50 nJ, 100 nJ, 150 nJ, 200 nJ, 500 nJ, 1000 nJ, 1500 nJ, and 2000 nJ is presented in Figure 5. The hot exciton cooling and auger recombination of the GSB signal is shown in Figure 5a and b, respectively. In Figure 5a, a cooling time of hundreds of fs for hot excitons can be observed. With the greater increment in pump–pulse energy, the depth of the GSB signal increases with the pump–pulse energy. In Figure 5b, as confirmed from Figure 4, when the pump–pulse energy is low, e.g., 100 nJ or lower, it is followed by a slow decay much longer than 1 ns, without the typical multi-exciton fast decay performance. With the increase in the pump–pulse energy to 150 nJ or higher, after tens of ps after the peak value, the signal quickly decays, showing a typical proportional relation between the decay rate and incident energy. When the pump–pulse energy reaches the threshold of multi-photon absorption, the multi-exciton experiences auger recombination at different speeds. At the early stage, the auger recombination plays the key role. Later, the multi-exciton transfers to a single exciton due to auger recombination, which generally leads to radioactive recombination with a slower decay rate.

To quantitatively explore the dependence of hot exciton cooling time and auger recombination rate on the pump–pulse energy in multi-photon absorption, for the GSB signal of the probe light at 470 nm evolution with the delay time between the probe and the pump light under various pump–pulse energies, exponential fitting curves were plotted in Figure 5a,b, which follows the relations in [35,36] as:

∆A(t) = A_1_exp(−t/τ_1_) + A_2_exp(−t/τ_2_) + A_3_exp(−t/τ_3_) − A_0_exp(−t/τ_e_) (4)

where A_i_, i = 0,1,2,3 is the amplitude of each term. Each amplitude A_i_ in Equation (4) represents the proportion of each physical process in the exciton generation and recombination process. A_0_ represents the coefficient of hot exciton cooling time before the exponential function is fitted during exciton generation. A_1_, A_2_, and A_3_ represent the coefficients of defect state filling, auger recombination, and radiation recombination, respectively, before the exponential function is fitted during exciton recombination. τ is the time constant in ps; τ_e_ is the delay of the electron transition from higher levels to the bottom level of the conduction band, i.e., the cooling time of hot excitons, which is approximately in the range of hundreds of fs. Compared to the fitting formulas in the references, the time decay constant τ_1_ is added to respond to the first rapid decay process of the GSB signal. It also represents the filling time of surface defects in QDs according to the photoluminescence spectrum analyses in Figure 3a [37]. τ_2_ represents the non-radioactive auger recombination around several ps to hundreds of ps, responding to the second rapid decay process of the GSB signal; τ_3_ represents the radioactive recombination on the order of ns. The fitted results are given in Table 1. The numbers in the second column of brackets represent the proportion of hot excitons cooling in the whole exciton generation process, and the ones in the third to fifth columns represent the proportions of defect filling, auger recombination process, and radiation recombination process in the whole exciton recombination process, respectively. The filling time of surface defect τ_1_ is only maintained for 10–20 ps, which shows a relatively stable value that is insignificant for either auger recombination or single-exciton radioactive recombination, so it is expressed in parentheses in the form of ‘/’. Additionally, due to the use of the same sample for testing, i.e., the same surface defect state, only the value of the pump–pulse energy was changed. When comparing the test results with each other, it can be considered that the difference in test results is due to the different rates of auger recombination between excitons. When using the 340 nm-wavelength pump light, the cooling time τ_e_ of hot excitons increases from 184 fs to 441 fs under the pump–pulse energy from 50 nJ to 2000 nJ. When the pump–pulse energy is 50 nJ and 100 nJ, formulas cannot be used to successfully fit the various time constants, which also indicates that no auger recombination occurs during exciton decay when pumping with a lower pump–pulse energy. When the pump–pulse energy increases from 150 nJ to 2000 nJ, the auger recombination time τ_2_ decreases from 101.09 ps to 61.41 ps. During the exciton decay process, the ratio of auger recombination in the material increases from 30.5% to 52.4%, while the ratio of single-exciton recombination decreases from 69.5% to 47.6%, which shows a coherent trend as published in PbS QDs for high-energy exciton dynamics [26]. As a result, when the CdSe QDs absorb sufficient energy to generate multi-exciton, with the increase in pump–pulse energy, the cooling time is extended while the auger recombination is accelerated and competes with the radioactive recombination. When the energy continues to increase, the multi-photon absorption will be further dominated. As given in our results, the start of multi-photon absorption from a single photon should be in the range of 100 to 150 nJ incident pump–pulse energy. After that, the radioactive recombination of excitons generally transfers to the auger recombination. The experimental exciton dynamics results of CdSe QDs can provide a reference for exciton dynamics research of other types of QDs.

## 4. Conclusions

In summary, the exciton dynamics of CdSe QDs is studied using a 340 nm-wavelength pump light based on a TA spectroscopy measurement. For a lower pump light pulse energy below 100 nJ, the exciton evolution is dominated by single-photon absorption and radioactive recombination. When the pump–pulse energy increases to higher than 150 nJ, multi-photon absorption with auger recombination begins and competes with the single-photon process. Due to the interaction force between excitons, the peak position of the GSB signal in the transient absorption spectrum will experience a red shift. With the increase in pump–pulse energy, the cooling time of hot excitons extends while the rate of auger recombination is accelerated. When the pump–pulse energy increases from 50 nJ to 2000 nJ, the cooling time increases from 184 fs to 441 fs. For radioactive recombination under low pump–pulse energies, the process lasts for the range of ns. For higher pulse energies above 150 nJ, the auger recombination decreases from 101.09 ps to 61.41 ps. The auger recombination in the material competes with the radioactive combination process, and increases from 30.5% under lower pump–pulse energy to 52.4% under strong laser power. In general, this research will help to understand the principle of multi-photons in QDs and explore new manufacturing solutions for QD optoelectronic devices.

## Figures and Tables

**Table 1:** Fitting parameters of the GSB kinetic curves of CdSe QDs.

Pump–Pulse Energy/nJ	τ_e_/ps (Weights/%)	τ_1_/ps (Weights/%)	τ_2_/ps (Weights/%)	τ_3_/ps (Weights/%)
50	0.184 (100)	/	/	/
100	0.228 (100)	/	/	/
150	0.246 (100)	20.56 (/)	101.09 (30.5)	>2 ns (69.5)
200	0.290 (100)	20.36 (/)	89.53 (39.8)	>2 ns (60.2)
500	0.311 (100)	19.74 (/)	87.08 (40.8)	>2 ns (59.2)
1000	0.368 (100)	13.07 (/)	80.29 (43.7)	>2 ns (56.3)
1500	0.416 (100)	15.19 (/)	72.61 (48.9)	>2 ns (51.1)
2000	0.441 (100)	18.45 (/)	61.41 (52.4)	>2 ns (47.6)

## Data Availability

The data generated and analyzed during this study are contained within the article.
